# An Improved Approach for Estimating Daily Net Radiation over the Heihe River Basin

**DOI:** 10.3390/s17010086

**Published:** 2017-01-04

**Authors:** Bingfang Wu, Shufu Liu, Weiwei Zhu, Nana Yan, Qiang Xing, Shen Tan

**Affiliations:** Institute of Remote Sensing and Digital Earth (RADI), Chinese Academy of Sciences, Beijing 100094, China; liusf01@radi.ac.cn (S.L.); zhuww@radi.ac.cn (W.Z.); yannn@radi.ac.cn (N.Y.); xingqiang@radi.ac.cn (Q.X.); tanshen@radi.ac.cn (S.T.)

**Keywords:** daily net radiation, sunshine duration, cloud classification, FY-2D, Heihe River Basin

## Abstract

Net radiation plays an essential role in determining the thermal conditions of the Earth’s surface and is an important parameter for the study of land-surface processes and global climate change. In this paper, an improved satellite-based approach to estimate the daily net radiation is presented, in which sunshine duration were derived from the geostationary meteorological satellite (FY-2D) cloud classification product, the monthly empirical *a_s_* and *b_s_* Angstrom coefficients for net shortwave radiation were calibrated by spatial fitting of the ground data from 1997 to 2006, and the daily net longwave radiation was calibrated with ground data from 2007 to 2010 over the Heihe River Basin in China. The estimated daily net radiation values were validated against ground data for 12 months in 2008 at four stations with different underlying surface types. The average coefficient of determination (*R*^2^) was 0.8489, and the averaged Nash-Sutcliffe equation (*NSE*) was 0.8356. The close agreement between the estimated daily net radiation and observations indicates that the proposed method is promising, especially given the comparison between the spatial distribution and the interpolation of sunshine duration. Potential applications include climate research, energy balance studies and the estimation of global evapotranspiration.

## 1. Introduction

Net radiation (*R_n_*) is the balance between the downward and upward shortwave and longwave radiation and is a key component of the Earth’s surface energy balance. It is the main source of energy for the physical and chemical processes that occur in the surface-atmosphere interface, the heat and water budgets, photosynthesis [1] and evapotranspiration [2], which are used as input for global and regional climate change and eco-hydrological models. Net radiation especially affects the energy balance closure and the accuracy of evapotranspiration estimation algorithms [3,4]; therefore, the accurate estimation of net radiation is important for researchers in the fields of meteorology, hydrology, global change and agriculture [5,6,7].

Net radiation can be reliably obtained using net radiometers or shortwave and longwave radiometers, and it is routinely recorded at meteorological and radiation stations. Although such instruments are accurate for measuring net radiation at a station (representative for a certain area), their use for large regional net radiation assessments is time consuming and expensive because numerous ground installations are required, especially when large spatial coverage and a high sampling frequency are desired. Net radiation is not measured directly in most national basic meteorological stations due to restrictions imposed by economic and technical conditions, unless the station is required for radiation studies. In addition, few national radiation stations exist, resulting in insufficient net radiation data in some areas.

Various methods have been recommended for obtaining net radiation on a regional scale. Currently, the most commonly used methods for calculating net radiation are applied to the calculation of net shortwave and net longwave radiation. Net shortwave radiation is obtained by using the surface albedo and global solar radiation. The surface albedo can be calculated from remote sensing data using different band reflectances [8]. Global solar radiation can be obtained by several empirical models involving various climatic variables, including extra-terrestrial solar radiation, sunshine duration, mean temperature, maximum temperature, minimum temperature, water saturation deficit, soil temperature, the number of rainy days, altitude, latitude, total precipitation, the Sun-Earth distance, and the Julian day [9,10,11,12,13,14,15,16,17,18,19,20,21,22,23,24,25,26,27,28,29,30,31,32,33,34,35,36,37]. The most widely used method is the Angstrom-Prescott model, which has been applied in many countries [24,38,39,40,41,42,43] in various forms, such as quadratic, third degree, logarithmic and exponential forms. The Angstrom-Prescott model and its revised variants most commonly use sunshine duration for estimating global solar radiation. According to the comparative study by Can and Oman [44] and Kadir [35], global solar radiation models based on sunshine duration could give more accurate results than those based on other meteorological variables without sunshine duration. Although sunshine duration can be obtained from ground measurements, their application to regional and global solar radiation calculations suffers from the limited availability of ground data. Robaa [45] developed an empirical method based on the amount of clouds to estimate sunshine duration, in which empirical equations were proposed based on relative sunshine duration and readily available cloud amount data from Moderate Resolution Imaging Spectroradiometer (MODIS). This method does not consider the changes in the cloud amount between sunrise and sunset, and it can calculate only monthly sunshine duration, not daily sunshine duration. Wu et al. [46] proposed a new method to derive sunshine duration from geostationary meteorological satellite hourly cloud classification data based on a new index—the cloud classification coverage-type sunshine factor—and this method can accurately estimate daily sunshine duration without ground measurements.

In addition to sunshine duration, the empirical Angstrom *a_s_* and *b_s_* coefficients are needed to calculate global solar radiation using the original and revised variants of the Angstrom-Prescott model. Common values of these coefficients range between 0 and 1 (0.25 and 0.5 have been recommended when no ground measurements are available for calibration). The former expresses the fraction of extra-terrestrial radiation reaching the ground on an overcast day (*n* = 0) and depends on the atmospheric conditions (humidity and aerosols) and the solar angle [9,35,47]. Ground measurements are commonly used to fit the values of *a_s_* and *b_s_*, and several researchers [36,48,49,50,51] have estimated these coefficients on an annual scale in different regions. However, using a fixed coefficient on an annual scale is not appropriate for our purposes because of the regional environmental characteristics, and thus, the monthly empirical Angstrom coefficient should be fitted. Zhao et al. [37] suggested a new approach based on Air Pollution Index (API) data from ground measurements to adjust the Angstrom coefficients. Because of the limited number of ground stations and the short site observation time, this method has high precision but is not generalizable. Therefore, ground-measured radiation and meteorological data can be fully utilized to fit the monthly empirical Angstrom coefficients *a_s_* and *b_s_*, which is a better method to estimate global solar radiation based on sunshine duration data.

Meteorological variables are commonly used to estimate net longwave radiation: water vapour pressure, air temperature, and downward shortwave radiation or sunshine duration, as in the Food and Agricultural Organization of the United Nations (FAO)-56 Penman equation [17,27,52]. The daily net longwave radiation equation coefficients based on the FAO-56 Penman equation have been applied to various situations in many countries and regions. However, it must be calibrated with local ground measurements [47,53,54,55], and the downward shortwave radiation and sunshine duration required in the calibration process can be calculated based on geostationary meteorological satellite hourly cloud classification data.

The objective of this study was to investigate an improved method to derive daily net radiation based on remote sensing products coupled with field measurement data. We focused on calculating global solar radiation based on sunshine duration from Feng-Yun (FY)-2D geostationary meteorological satellite data using the method described by Wu et al. [46], monthly *a_s_* and *b_s_* Angstrom coefficients based data from radiation stations, and the calibration of daily net longwave radiation based on ground measurements. The estimated daily net radiation values from this proposed algorithm were validated with independent ground observation data, and the spatial distribution was compared with the results of net radiation based on the sunshine duration using meteorological station data interpolation.

## 2. Methodology

### 2.1. Study Area

**Figure 1:**
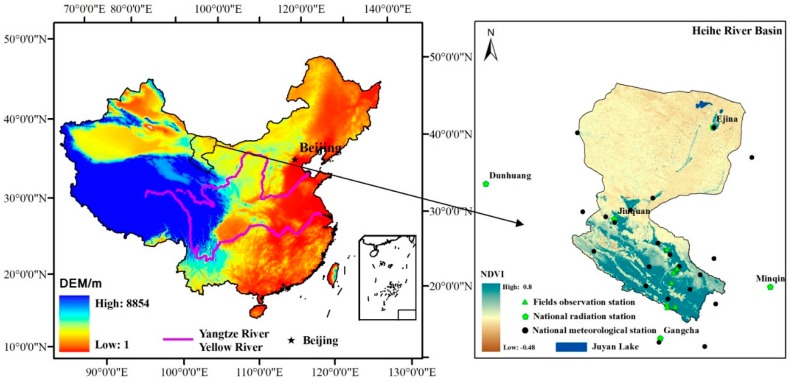
Location and physiography of the Heihe River Basin.

The Heihe River Basin, located in the northwest region of China between 97°24′ E–102°10′ E and 37°41′ N–42°42′ N, is the second largest inland river basin in China, covering an area of approximately 143,000 km^2^. Its elevation ranges from approximately 5000 m in the upper reaches to 1000 m downstream. The river originates in the Qilian mountains and flows through the Hexi corridor of Gansu Province from the Yingluo Gorge, through the Zhengyi Gorge, and then northward into the Ejina oasis in the western part of the Inner-Mongolia Plateau, before finally discharging into the east and west Juyan lakes (Figure 1). The landscape varies from glaciers and frozen soil to alpine meadow, forest, irrigated cropland, riparian ecosystems, bare Gobi, and desert. A large proportion of the watershed area comprises sparse vegetation or bare land. The highest air temperatures are approximately 40 °C downstream in the summer, and the lowest are approximately −40 °C in the upper watershed in winter. The mean annual rainfall across the basin is 110 mm·year^−1^ (1980–2010) [56], and the annual precipitation in the upstream area is more than 350 mm·year^−1^, it is 100–250 mm·year^−1^ in the middle reaches, and the annual precipitation in the downstream area is less than 50 mm·year^−1^.

Because of the scarce water resources in this basin, a large number of hydrology and water resources, environmental ecology, and land surface process research projects have been conducted in the region since 1989, such as Heihe Basin Field Experiment (HEIFE) [57,58], Arid Environment Comprehensive Monitoring Plan ’95 (AECMP ’95) [59], DunHuang Experiment (DHEX) [60], the Watershed Allied Telemetry Experimental Research (WATER) project [61], and the Heihe WATER (HiWATER) project [62]. A large body of ground observation data from meteorological, hydrological and energy flux stations and associated study results has been accumulated. This rich knowledge resource has laid the foundation for much of the scientific research in the Heihe River Basin, and also the high heterogeneity of the underlying surface and the strong seasonal weather changes in the Heihe River Basin can better test the feasibility of the new method proposed in this paper.

### 2.2. Data and Process

#### 2.2.1. Field Observation Data

The field observation data include radiation and meteorological data obtained during the WATER experiments [61], which can be downloaded from http://westdc.westgis.ac.cn. The observation stations involved in the WATER experiments are described in Table 1. Yingke station was installed in the central area of the oasis, which is located within typical irrigated farmland on which maize and wheat are grown. Linze station was installed in the grassland ecological experimental station of Lanzhou University in Linze County on typical wetland and saline alkali land. Huazhaizi station was installed on the desert beach in the southern city of Zhangye in a typical bare Gobi landscape in the middle reaches of the Heihe River Basin. Arou, Yakou, Binggou and Maliantan stations are representative of the upper reaches of the Heihe River Basin, which consist of typical alpine meadow and sparse grass. An automatic meteorological observation system and a radiation observation system were installed at each site to obtain the air temperature, air humidity, wind speed, wind direction, air pressure, precipitation, soil temperature and the soil moisture profile, solar radiation, upward short-wave radiation, upward long-wave radiation, downward long-wave radiation and soil heat flux data. Quality control for all ground measurements was performed by the data suppliers. Observation data from Yakou, Binggou and Huazhaizi stations from 2007 to 2010 and from Yingke, Linze, Arou and Maliantan stations in 2007, 2009 and 2010 were used to calibrate the parameters of the proposed net longwave radiation model. The remaining 2008 observations from Yingke, Linze, Arou and Maliantan stations were used for model validation.

#### 2.2.2. MODIS and FY-2D Satellite Data

The MODIS 1B clear-sky data covering the Heihe River Basin were collected for 2007 to 2010. We performed a geometric correction using the built-in Ground Control Point (GCP) of the image and calculated the reflectance or radiance, which provided the calibration coefficients. We determined the cloud pixels using a method proposed by Ackerman et al. [63] that separates clear pixels from cloud-contaminated pixels using threshold values with multiple characteristics. The normalized difference vegetation index (NDVI) in the experimental area was calculated using the MODIS 1 2-band reflectivity after atmospheric correction, and the leaf area index (LAI) was calculated from the NDVI [64]. The surface albedo was computed based on a linear combination of the first seven reflectance bands [8].

FY-2D cloud data were generated by the FY-2D geostationary meteorological satellite at a resolution of 5 km [65,66]. Hourly FY-2D cloud classification products covering the Heihe River Basin from 2007 to 2010 were obtained (35,064 images) from the China Meteorological Administration (CMA) in Hierarchical Data Format (HDF). The hourly cloud classification coverage-type (cloud-type) data for the Heihe River Basin in a geographic projection were obtained based on a geographic lookup table (GLT) file downloaded from the National Satellite Meteorological Center (NSMC) [46].

#### 2.2.3. Meteorological and Radiation Data from National Stations

Daily radiation data from the national radiation stations (Figure 1 and Table 2) covering the Heihe River Basin and the surrounding area from 1997 to 2006 were provided by the China National Meteorological Bureau and included sunshine duration, solar irradiation, net radiation, diffuse irradiation, horizontal direct beam irradiation, reflected radiation and vertical direct beam irradiation.

Daily meteorological data from 21 meteorological stations (Figure 1) covering the Heihe River Basin and the surrounding area from 2007 to 2010 were also provided by the China National Meteorological Bureau, including the maximum air temperature, minimum temperature and air humidity. To calculate the net radiation, all variables were interpolated into a daily map at a resolution of 1 km. Squared inverse distance weighting was used for air temperature interpolation, in combination with digital elevation model (DEM) data, and thin plate splines were employed for air humidity interpolation [67].

All the daily radiation and meteorological data used in this paper were provided by the China Meteorological Data Center and can be downloaded from http://cdc.cma.gov.cn/cdc_en/home.dd. Quality control of the data was performed by the suppliers.

### 2.3. Modelling Daily Net Radiation

The energy balance of a land surface can be described as:

(1) $$R_{n}=R_{sd}-R_{su}+R_{ld}-R_{lu}$$

where *R_n_* is the net radiation (MJ·m^−2^·d^−1^), *R_sd_* is the downwelling shortwave radiation (MJ·m^−2^·d^−1^), *R_su_* is the upwelling shortwave radiation (MJ·m^−2^·d^−1^), *R_ld_* is the downwelling longwave radiation (MJ·m^−2^·d^−1^), and *R_lu_* is the upwelling longwave radiation (MJ·m^−2^·d^−1^). Alternatively, *R_n_* is the sum of the daily net shortwave radiation and the daily net longwave radiation and can be written as:

(2) $$R_{n}=R_{ns}+R_{nl}$$

where *R_ns_* is the net shortwave radiation (MJ·m^−2^·d^−1^), and *R_nl_* is the net longwave radiation (MJ·m^−2^·d^−1^). The net shortwave radiation can be calculated from the global solar irradiation (*R_s_*) and the surface albedo (*α*) as:

(3) $$R_{ns}=R_{s}\left(1-\alpha \right)$$

The surface albedo can be computed from a linear combination of the first seven reflectance bands based on MODIS data. If the global solar irradiation and daily net longwave radiation are known, the daily net radiation can be estimated on a regional scale by combining Equations (2) and (3).

#### 2.3.1. Global Solar Radiation

The most widely used method to estimate global solar radiation is the Angstrom-Prescott model [9,68,69]:

(4) $$R_{s}=\left(a_{s}+b_{s}\frac{n}{N}\right)R_{a}$$

where *R_s_* and *R_a_* are the global solar radiance (MJ·m^−2^·d^−1^) and extra-terrestrial solar irradiance (MJ·m^−2^·d^−1^), respectively; *n* stands for the actual sunshine duration of bright sunshine (*h*); *N* is the maximum possible sunshine duration (*h*); and *a_s_* and *b_s_* are empirically determined regression coefficients.

The following equation combines the new index cloud-type factor [46], which is shown in Table 3. The hourly FY-2D cloud type product mentioned in Section 2.2.2 as used to estimate the daily sunshine duration (*n*) with a resolution of 1 km over the Heihe River Basin:

(5) $$FY_{sunt}=\sum_{i=h_{sr}+0.25}^{i=h_{ss}-0.25}SF_{i}\times T_{gap}$$

where *FY_sunt_* stands for the sunshine duration (between 15 min (+0.25 h) after the start of sunrise and 15 min (−0.25 h) before sunset and the accumulation of sunshine factors); SF is the FY-2D cloud cover-type factor (Table 3), which is the index for the hourly FY-2D hourly cloud type data from sunrise to sunset; *T_gap_* is an hour’s interval with a value of 1; *i* is the time series that ranges between sunrise and sunset at the local time; and *h_sr_* and *h_ss_* are the times of sunrise time and sunset, respectively, and can be calculated from the latitude and solar declination based on the day of the year.

The extra-terrestrial solar irradiance *R_a_* (MJ·m^−2^·d^−1^) is calculated as follows:

(6) $$R_{a}=\frac{24\times 60}{\pi }G_{sc}d_{r}\left[\omega_{s}\sin\left(\varphi \right)\sin\left(\delta \right)+\cos\left(\varphi \right)\cos\left(\delta \right)\sin\left(\omega_{s}\right)\right]$$

where *G_sc_* is the solar constant (0.082 MJ·m^−2^·min^−1^), *d_r_* is the inverse relative distance between the Earth and the Sun, *φ* is the latitude (radians), *δ* is the solar declination (radians), and *ω_s_* is the sunset hour angle (radians). The values of *d_r_* and *δ* can be calculated with Equations (7) and (8):

(7) $$d_{r}=1+0.033\cos(\frac{2\pi }{365}J)$$

(8) $$\delta =0.409\sin\left(\frac{2\pi }{365}J-1.39\right)$$

where *J* is the Julian day (the day of the year) between 1 January and 31 December (*J* = 1, …, *n*; *n* = 365 or 366). The parameter *ω_s_* is calculated with *φ* and *δ* as:

(9) $$\omega_{s}=\arccos[-\tan\left(\varphi \right)\tan\left(\delta \right)]$$

The maximum possible sunshine duration, *N* in Equation (4), is calculated as follows:

(10) $$N=\frac{24}{\pi }\omega_{s}$$

Based on the actual sunshine duration from the hourly FY-2D cloud type and Equation (5), Equation (4) can be used to calculate the regional daily global solar radiation over the Heihe River Basin, provided the regression *a_s_* and *b_s_* Angstrom coefficients are known. Daily radiation records (1997–2006) for the stations in Table 2 were used to compute the Angstrom coefficients, which were subsequently spatially interpolated and combined with daily sunshine duration from the hourly FY-2D cloud type based on the Equation (5).

#### 2.3.2. Net Longwave Radiation

When using the FAO-56 Penman method, the net longwave radiation (*R_nl_*, MJ·m^−2^·d^−1^) is calculated by determining the net longwave radiation for a clear sky, which has been improved by various researchers [47,53,70,71] for cloud cover conditions. The equation can be written as:

(11) $$R_{nl}=\sigma \frac{T_{\max}^{4}+T_{\min}^{4}}{2}\varepsilon_{net}f_{cloudiness}$$

where *σ* is the Stefan-Boltzmann constant (MJ·m^−2^·d^−1^·K^−4^); $T_{\max}^{4}$ and $T_{\min}^{4}$ are the daily maximum and daily minimum air temperatures at a height of 2 m (K), respectively; *ε_net_* is the net emissivity; and *f_cloudiness_* is the cloudiness factor. The value of *ε_net_* is calculated as the emissivity of vegetation (*ε_vs_*) minus the atmospheric emissivity (*ε_a_*) using the following equation:

(12) $$\varepsilon_{net}=\varepsilon_{vs}-\varepsilon_{a}$$

The factor *ε_vs_* is commonly calculated using the *LAI*. According to Bastiaanssen [72], the formula used for this calculation is as follows:

(13) $$\varepsilon_{vs}=0.95+0.01LAI,\;\mathrm{when}\;LAI\;<3$$

(14) $$\varepsilon_{vs}=0.98,\;\mathrm{when}\;LAI\;\geq 3$$

The value of *ε_a_* is obtained by applying the Brunt equation [70]:

(15) $$\varepsilon_{a}=a_{1}+b_{1}\sqrt{e_{a}}$$

where *a*_1_ and *b*_1_ are coefficients, and *e_a_* is the water vapour pressure (kPa). The cloudiness factor is [52]:

(16) $$f_{cloudiness}=c_{1}\frac{n}{N}+d_{1}$$

or:

(17) $$f_{cloudiness}=c_{2}\frac{R_{s}}{R_{so}}+d_{2}$$

with:

(18) $$R_{so}=\left(a_{s}+b_{s}\right)R_{a}$$

(19) $$c_{2}=c_{1}\times \frac{a_{s}+b_{s}}{b_{s}}$$

(20) $$d_{2}=-c_{1}\times \frac{a_{s}}{b_{s}}+d_{1}$$

where *c*_1_, *c*_2_, *d*_1_ and *d*_2_ are coefficients, and *R_so_* is the clear sky solar radiation (MJ·m^−2^·d^−1^). Penman [71] and Jensen [53] suggest that *c*_1_ = 0.9 and *d*_1_ = 0.1. Allen [47] applied Equation (17) with *c*_2_ = 1.35 and *d*_2_ = −0.35, which were obtained by substituting the empirical *a_s_* and *b_s_* coefficients (*a_s_* = 0.25 and *b_s_* = 0.50) into Equation (4) and Equation (18) into Equation (16) using the values of empirical coefficients suggested by Penman [71] and Jensen [53].

In this study, Equation (17) has been adopted for the cloudiness factor. The empirical coefficients (*c*_2_ and *d*_2_) are fitting parameters, which are obtained using Equations (11)–(20) with ground observations from the Yakou, Binggou and Huazhaizi stations from 2007 to 2010 and the Yingke, Linze, Arou and Maliantan stations in 2007, 2009 and 2010. The values of *a_s_* and *b_s_* were obtained as indicated in Section 2.3.1. The optimal sum of *c*_2_ and *d*_2_ is 1.0.

In this paper, the three calibration methods described by Hiroyuki [52] were used to calibrate the parameters *a*_1_, *b*_1_, *c*_2_ and *d*_2_. First, the two coefficients (*a*_1_ and *b*_1_) in Equation (15) are calibrated to minimize the root-mean-square error (*RMSE*) of the estimated longwave downward radiation when the relative sunshine duration value exceeds 0.95 (step 1). Then, the values of coefficients *c*_2_ and *d*_2_ were calculated by Equations (19) and (20) using the values of *c*_1_ and *d*_1_ suggested by Penman [71] and Jensen [53] (step 2). Finally, in step 3, *c*_2_ and *d*_2_ in Equation (17) were directly calibrated using the coefficients obtained in step 1 (*a*_1_ and *b*_1_) and the estimated values of *a_s_* and *b_s_* from Section 2.3.1 to minimize the *RMSE* of the estimated daily net longwave radiation with respect to the observed records. If the *a*_1_, *b*_1_, *c*_1_, *c*_2_, *d*_1_ and *d*_2_ coefficients are known, net longwave radiation can be estimated on a regional scale by combining Equations (11)–(20).

### 2.4. Model Performance Assessment

The model parameters are determined using numerical iteration methods [73]. The statistical indices used to evaluate the model performance include the coefficient of determination (*R*^2^), the mean bias error (*MBE*), the mean absolute error (*MAE*), the *RMSE*, the index of agreement (*d*), and the Nash-Sutcliffe Equation (*NSE*) [74,75]. These values are defined as follows:

(21) $$R^{2}=\frac{{\left[\sum_{i=1}^{n}\left(O_{i}-\bar{O}\right)\left(P_{i}-\bar{P}\right)\right]}^{2}}{\sum_{i=1}^{n}{\left(O_{i}-\bar{O}\right)}^{2}{\left(P_{i}-\bar{P}\right)}^{2}}$$

(22) $$\mathrm{MBE}=n^{-1}\sum_{i=1}^{n}\left(P_{i}-O_{i}\right)$$

(23) $$\mathrm{MAE}=n^{-1}\sum_{i=1}^{n}\left|P_{i}-O_{i}\right|$$

(24) $$\mathrm{RMSE}=\sqrt{n^{-1}\sum_{i=1}^{n}{\left(P_{i}-O_{i}\right)}^{2}}$$

(25) $$d=1-\frac{\sum_{i=1}^{n}{\left(P_{i}-O_{i}\right)}^{2}}{\sum_{i=1}^{n}{\left(\left|P_{i}-\bar{O}\right|+\left|O_{i}-\bar{O}\right|\right)}^{2}}$$

(26) $$\mathrm{NSE}=1-\frac{\sum_{i=1}^{n}{\left(O_{i}-P_{i}\right)}^{2}}{\sum_{i=1}^{n}{\left(O_{i}-{\bar{O}}_{i}\right)}^{2}}$$

where *O_i_* is the actual measurement, and *P_i_* is its estimate; $\bar{O}$ is the mean measurement; $\bar{P}$ is the mean of the estimates; and *n* is the sample size. The ideal values of the statistical tests, such as the *MAE* and *RMSE*, are 0 or close to 0. A model is more efficient when the *NSE*, *R*^2^ and *d* values are closer to 1 [76]. Fox [77] and Willmott [78] also suggest the following: (1) The model should perform well when the *MAE* is less than 50% of the measured standard deviation; and (2) higher values of *d* are correlated with better model performance.

## 3. Results

### 3.1. Monthly Angstrom Coefficients

The monthly Angstrom coefficients *a_s_* and *b_s_* at five national radiation stations were estimated as the regression coefficients of the long-term monthly average radiation and sunshine duration (1997–2006) per Equation (4), and the results are listed in Table 4. The highest coefficient of determination (*R*^2^) between the estimated solar radiation based on those regression monthly Angstrom coefficients and the observed solar radiation is 0.98, and the averaged value is 0.93. The average values of the coefficients *a_s_* and *b_s_* are 0.23 and 0.53, respectively; *a_s_* varies between 0.16 and 0.36, while *b_s_* varies from 0.44 to 0.62. The average value of *a_s_* is less than and that of *b_s_* is larger than the values reported by Angstrom [9] and Chen [32]. The above results indicate no obvious difference in the accuracy of the estimation of solar radiation in the low-latitude and high-latitude areas based on the monthly Angstrom coefficients. According to the monthly Angstrom coefficients at each station, the spatially distributed *a_s_* and *b_s_* can be obtained by spatial interpolation (inverse distance weighting with exponent 2).

### 3.2. Validation of the Global Solar Radiation

The annual Angstrom coefficients *a_s_* and *b_s_* at five national radiation stations were also estimated as the regression coefficients of the long-term annual average radiation and sunshine duration (1997–2006) per Equation (4). The same spatial interpolation method (inverse distance weighting with exponent 2) was used to obtained the spatially distributed of annual *a_s_* and *b_s_* based on the annual Angstrom coefficients data at each station.

**Figure 2:**
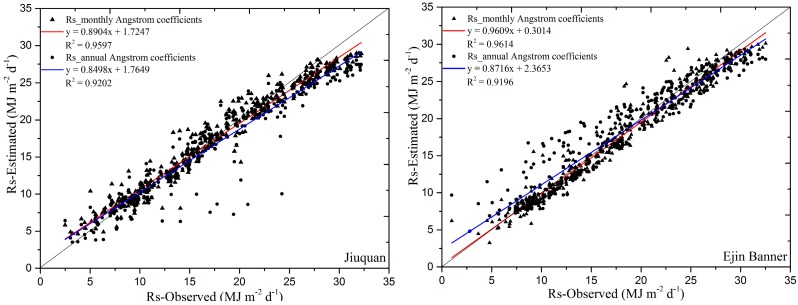
Comparison of the observed and estimated global daily solar radiation in 2008 (366 days) at Jiuquan (**left**) and Ejin Banner (**right**).

Two typical national radiation stations were randomly selected. The ground measurement data from these two stations in 2008 (366 days) were used to compare the estimates of daily global solar radiation determined using Equation (4) based on the spatial data of the monthly Angstrom coefficients and annual Angstrom coefficients, and also daily sunshine duration calculated from hourly FY-2D cloud-type data. Table 5 and Figure 2 show the validation results for the estimation of the daily global solar radiation and the ground measurement global solar radiation at two national radiation stations in 2008. Compared to the validation results between estimated global daily solar radiation based on the annual Angstrom coefficients and the observed values, the coefficients of determination (*R*^2^) between the observed and estimated values from monthly Angstrom coefficients have a higher value, which were 0.9597 at Jiuquan and 0.9614 at Ejin Banner, indicating more strong correlation between the observed daily global solar radiation and the estimated daily global solar radiation from monthly Angstrom coefficients; the index of agreement (*d*) and *NSE* were 0.9873 and 0.9541 at Jiuquan and 0.9894 and 0.9588 at Ejin Banner, respectively. The higher *d* and *NSE* indicate that daily global solar radiation estimated by the proposed method correlates better with the in situ daily global solar radiation. The *MBE*, *MAE* and *RMSE* at these two stations were −0.1849 MJ·m^−2^·d^−1^, 1.2432 MJ·m^−2^·d^−1^, 1.6419 MJ·m^−2^·d^−1^, and −0.3860 MJ·m^−2^·d^−1^, 1.1291 MJ·m^−2^·d^−1^, 1.4946 MJ·m^−2^·d^−1^, respectively. The relativized magnitude of *MBE* is from 5.9945 MJ·m^−2^·d^−1^ to 5.3658 MJ·m^−2^·d^−1^ at Jiuquan and from −3.1381 MJ·m^−2^·d^−1^ to 6.3227 MJ·m^−2^·d^−1^ at Ejin Banner. The lower *MAE* and *RMSE* values between the observed and the estimated values by the proposed method than the results by the annual Angstrom coefficients suggest that few outliers are present in the estimated daily global solar radiation values.

### 3.3. Calibration of Net Longwave Radiation

All the observations from the Yakou, Binggou and Huazhaizi stations and the data from the Yingke, Linze, Arou and Maliantan stations in 2007, 2009 and 2010 were used to calibrate the model parameters of the proposed net longwave radiation model in Section 2.3.2. The calibration data encompassed different underlying surface types (upstream and midstream) in the Heihe River Basin. Based on Section 2.3.2, the final calibration coefficients show that *a*_1_, *b*_1_, *c*_1_, *c*_2_, *d*_1_ and *d*_2_ are 0.62, 0.15, 0.58, 0.84, 0.41 and 0.15, respectively. According to Equations (27) and (28), the final daily net longwave radiation equation after calibration and the comprehensive coefficients for the Heihe River Basin: 0.33 + 0.01*LAI* or 0.36, −0.15, 0.84 and 0.15, respectively; these values are substantially different from those suggested by Allen [47] (0.34, −0.14, 1.35 and −0.35, respectively) and Huang et al. [50] (0.39, 0.058, 0.1, and 0.9, respectively):

(27) $$R_{nl}=\sigma \left(\frac{T_{\max}^{4}+T_{\min}^{4}}{2}\right)\left(0.33+0.01LAI-0.15\sqrt{e_{a}}\right)\left(0.84\frac{R_{s}}{R_{so}}+0.15\right),\;\mathrm{for}\;LAI\;<3$$

(28) $$R_{nl}=\sigma \left(\frac{T_{\max}^{4}+T_{\min}^{4}}{2}\right)\left(0.36-0.15\sqrt{e_{a}}\right)\left(0.84\frac{R_{s}}{R_{so}}+0.15\right),\;\mathrm{for}\;LAI\;\geq 3$$

### 3.4. Net Radiation over the Heihe River Basin

**Figure 3:**
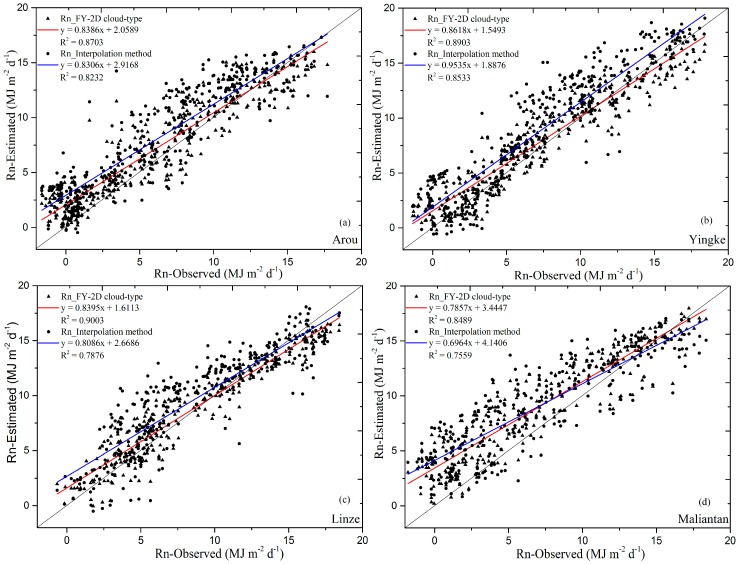
Comparison of the observed and estimated daily net radiation in 2008 (366 days) at Arou (**a**); Yingke (**b**) Linze (**c**) and Maliantan (**d**) stations.

After developing the daily solar radiation method and the calibration of the daily longwave radiation, the improved net radiation equation and the final parameters can be obtained. The 2008 ground data were used to validate the estimated daily net radiation. At the same time, an inverse distance weighting (IDW) interpolation method [79] was used to estimate daily sunshine duration, which was then applied to estimate the daily net radiation based on the method proposed in this paper instead of the sunshine duration calculated by the hourly FY-2D cloud-type data. The performance of the proposed method based on the sunshine duration from hourly FY-2D cloud-type data and station interpolation method is presented in Figure 3 and Table 6. Compared to the validation results between estimated daily net radiation based on the sunshine duration from station interpolation method (Rn_Interpolation method) and the observed values, the coefficients of determination (*R*^2^) between the observed values and the estimated values based on the sunshine duration from hourly FY-2D cloud-type data (Rn_FY-2D cloud-type) have a higher value, which were 0.8703, 0.8903, 0.9003 and 0.8489 at Arou, Yingke, Linze and Maliantan, respectively. The maximum *R*^2^ value is 0.9003, and the average is 0.8489, indicating a strong correlation between the observed and estimated values (Rn_FY-2D cloud-type). The indexes of agreement (*d*) at Arou, Yingke, Linze and Maliantan are 0.9518, 0.9684, 0.9771 and 0.9231, respectively, which also suggest good performance again. The *NSE* values at Arou, Yingke, Linze and Maliantan are 0.8279, 0.8837, 0.9160 and 0.7147, respectively. The maximum *NSE* value is 0.9160, and the average value is 0.8356. The higher values of *d* and *NSE* again indicate that the daily net radiation estimated by the proposed method (Rn_FY-2D cloud-type) correlates well with the in situ daily net radiation. The *MBE*, *MAE* and *RMSE* values determined at these four stations show that lower *MAE* and *RMSE* values from Rn_FY-2D cloud-type than the results by the Rn_Interpolation method are correlated with the existence of fewer outliers in the estimated daily net radiation values.

**Figure 4:**
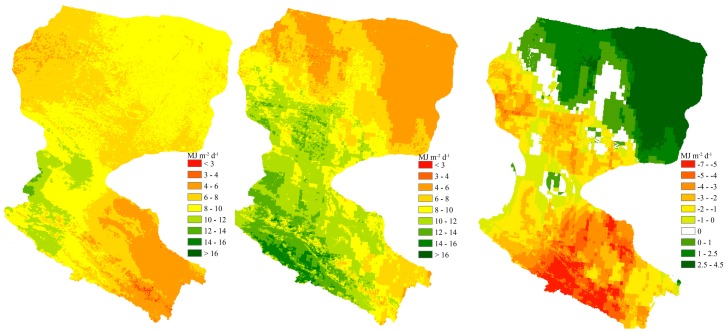
Spatial distribution of daily net radiation (MJ·m^−2^·d^−1^) estimated using sunshine duration-IDW (**left**); the proposed model (**middle**) and the difference between both methods (**right**) over the Heihe River Basin in 16 July 2008.

To explore the variation of the spatial distribution, Figure 4 compares the results for the Heihe River Basin on 16 July 2008 using the proposed method based on the sunshine duration from hourly FY-2D cloud-type data and station interpolation method. It’s also gave the spatial distribution of the difference between both methods in the Figure 4. Because meteorological stations are sparse in the upstream and downstream regions in Heihe River Basin, the difference between the two methods is obvious. Wu et al. [46] had described the spatial distribution of the sunshine duration based on the hourly FY-2D cloud-type data, in which the spatial distribution was better than by a method based on interpolation among meteorological stations data, especially in the upstream and downstream regions in Heihe River Basin due to few ground observation stations. Therefore, the method proposed in this paper based on the hourly FY-2D cloud-type data is clearly the best method to reflect the variation of the spatial distribution of the daily net radiation.

## 4. Discussion

Daily net radiation plays an essential role in determining the thermal conditions of the Earth’s surface. It is an important variable in modelling studies of land-surface processes and global climate change. The goal of this study was to accurately and easily estimate daily net radiation based on remote sensing products coupled with field measured data. This new method has the potential to estimate daily variations of surface net radiation and evapotranspiration over large regions.

Equation (4) indicates that a positive relationship exists between sunshine duration and global solar radiation. Additionally, the accuracy of the sunshine duration calculation directly affects the accuracy of the net radiation obtained by combining Equations (2), (27) and (28). The method based on the hourly FY-2D cloud type used to estimate sunshine duration is different from the interpolation method used for the ground measurement data, and it can better express the spatial variation of sunshine duration on a regional scale without ground measurements. For the downstream Heihe River Basin in particular, because of the lack of ground observation stations indicated in Figure 1, sunshine duration based on the hourly FY-2D cloud type can significantly improve the estimation of net radiation on a spatial scale, as shown in Figure 4.

The Angstrom coefficients *a_s_* and *b_s_* affect the solar radiation and net radiation based on Equations (2) and (4). Because of complex changes in the atmospheric conditions (humidity and aerosols) and the solar angle, an annual coefficient does not appropriately reflect the complex atmospheric conditions, and because of the limitations of the ground measurement data, it is impossible to obtain the daily Angstrom coefficients *a_s_* and *b_s_*. Therefore, in this study, the monthly Angstrom coefficients were calculated instead, as shown in Table 4, and better reflect the changes in the regional atmospheric conditions used to estimate the daily global solar radiation. In addition, the estimate of global solar radiation based on the spatial interpolation of the monthly Angstrom coefficients *a_s_* and *b_s_* from the ground sites was also better than that for the solar radiation based on a fixed coefficient on an annual scale, regardless of the spatial interpolation method applied.

Currently, various researchers are using different net longwave radiation estimation methods, all of which must be re-calibrated when applied to a new study site. In this paper, a set of parameters for the Heihe River Basin was calibrated for calculating net longwave radiation combined with LAI data and field measurement data. The parameter values were slightly different from those of Allen et al. [47] and Huang et al. [50] and could improve the net longwave radiation results.

Regarding the sunshine duration in mountain areas determined based on the Wu’s method using hourly FY-2D cloud-type data [46], because of the effects of topography and the heterogeneity of the mountain surface, the accuracy of the sunshine duration in the mountain area was less than that in the plain area. While more human activity occurs in the lowlands than in the mountains, the modification of atmospheric environment conditions affected by human activities in the lowlands was more frequently, so the monthly Angstrom coefficients *a_s_* and *b_s_* may also change in other years in the plain area even if the higher accuracy of the monthly Angstrom coefficients *a_s_* and *b_s_* have been obtained based on the historical data. Therefore, based on the Angstrom coefficients and sunshine duration, no obvious difference in the estimation accuracy of the net radiation between the mountain and plain areas was noted from Figure 3 and Table 6.

Figure 4 compares the results of the proposed method and that based on meteorological data interpolation for the Heihe River Basin. An obvious difference between the two methods for estimating the spatial distribution of net radiation was noted. Wu et al. [46] has described the spatial distribution of the sunshine duration based on the hourly FY-2D cloud-type data, in which the spatial distribution was better than by a method based on interpolation among meteorological stations data. While the proposed method does not depend on the number of surface weather stations, the monthly Angstrom coefficients of *a_s_* and *b_s_* were used instead of fixed coefficients, and a set of parameters was calibrated to calculate net longwave radiation combined with LAI data and field measurement data over the Heihe River Basin. Therefore, as shown in Figure 4 and Table 6, the method proposed in this paper can better reflect the difference in the spatial distribution of daily net radiation, especially in the upstream and downstream regions.

The proposed method allows daily net radiation to be derived from remote sensing products. For cloud-type data from FY-2D, the pixel value represents the average sunshine duration for an area of 5 km × 5 km. In addition, the cloud-type data do not account for smaller clouds, which also affect the sunshine duration estimation and daily net radiation. Ground measurements of sunshine duration and net radiation represent a much smaller area, which does not correspond to the area covered by a pixel. Consequently, some outliers would be expected in Table 6 and Figure 3, in which the estimated values are compared with the measured values. However, the terrain near most ground measurement stations is relatively flat with fairly homogeneous surface coverage. Thus, the ground measurement values can be assumed to represent the average value for the surrounding region and be appropriately matched with remotely sensed pixel values.

Whether fitting the monthly angstrom coefficients *a_s_* and *b_s_*, calibrating net longwave radiation parameters, or optimizing the FY-2D cloud-type factors, the proposed method requires a large quantity of ground data. Therefore, calculating the net radiation on a regional scale in the absence of ground data is difficult. Fortunately, data from reanalysis products (National Centers for Environmental Prediction [NCEP], Modern Era Retrospective Analysis for Research and Applications [MERRA] and Global Land Data Assimilation System [GLDAS]) can provide hourly, 3-h, 6-h or daily radiation and meteorological data. Thus, data from reanalysis products can be used in the proposed method to replace ground data for the fitting and calibration steps to estimate net radiation on a regional scale, even if the data from those reanalysis products are of low resolution.

The accuracy of net radiation estimates, net longwave radiation calibration and the Angstrom coefficients is negatively affected by the limited number of ground stations that measure radiation and other variables. More ground data or data from reanalysis products will be needed for model fitting and calibration to apply the proposed method in other regions.

## 5. Conclusions

A method was developed to derive daily net radiation based on remote sensing products coupled with field measurement data. The methodology was validated with data collected over the Heihe River Basin in northeast China in 2008. This method estimates daily global solar radiation based on sunshine duration from the FY-2D geostationary meteorological satellite, as described by Wu et al. [46]. A spatial fitting method was used to estimate the monthly *a_s_* and *b_s_* Angstrom coefficients based on ground radiation and meteorological data. The daily net longwave radiation was calibrated using ground data and the net longwave radiation equation. The results from this method show that the estimated daily net radiation and in situ measurements agree fairly well, confirming the soundness and good performance of the proposed method. The analysis of the spatial distribution of daily net radiation shows that the new method represents sunshine fields better than the interpolation of sunshine data.

## Figures and Tables

**Table 1:** Field observation station information for the Heihe River Basin.

Station Name	Longitude (°, E)	Latitude (°, N)	Elevation (m)	Land Cover	Observation Period	Location
Huazhaizi	100.32	38.77	1731	Bare Gobi	2008.06–2010.12	Midstream
Yingke	100.41	38.86	1519	Maize	2007.11–2010.12	Midstream
Arou	100.46	38.04	3033	Alpine meadow	2007.07–2010.12	Upstream
Linze	100.07	39.25	1394	Grass	2007.10–2008.10	Midstream
Maliantan	100.30	38.55	2817	Sparse grass	2007.11–2009.12	Upstream
Yakou	100.24	38.01	4147	Alpine meadow	2007.10–2009.10	Upstream
Binggou	100.22	38.07	3449	Sparse grass	2007.9–2009.9	Upstream

**Table 2:** National radiation station information for the Heihe River Basin.

Station Name	Longitude (°, E)	Latitude (°, N)	Elevation (m)	Land Cover	Observation Period	Location
Ejin Banner	101.07	41.95	941	Sparse forests	1997–2006	Downstream
Dunhuang	94.68	40.15	1139	Sparse forests	1997–2006	Midstream (outside)
Jiuquan	98.48	39.77	1477	Farmland	1997–2006	Midstream
Minqin	103.08	38.63	1367	Urban	1997–2006	Midstream
Gangcha	100.13	37.33	3302	Bare	1997–2006	Upstream (outside)

**Table 3:** FY-2D cloud-type factor for the Heihe River Basin.

Code	Cloud Type (Cloud Classification)	Factor
0/1	Clear Sky	0.9
11	Mixed pixels	0.21
12	Altostratus or Nimbostratus	0.25
13	Cirrostratus	0.51
14	Cirrus spissatus	0.24
15	Cumulonimbus	0.13
21	Stratocumulus or Altocumulus	0.35

**Table 4:** Monthly regression Angstrom coefficients of *a_s_* and *b_s_* at five national radiation stations.

Station	Month	*R*^2^	*a_s_*	*b_s_*	Station	Month	*R*^2^	*a_s_*	*b_s_*
Ejin Banner	1	0.88	0.27	0.49	Jiuquan	7	0.98	0.21	0.47
Ejin Banner	2	0.96	0.28	0.47	Jiuquan	8	0.94	0.23	0.50
Ejin Banner	3	0.94	0.25	0.45	Jiuquan	9	0.97	0.24	0.56
Ejin Banner	4	0.95	0.23	0.51	Jiuquan	10	0.92	0.22	0.52
Ejin Banner	5	0.96	0.25	0.55	Jiuquan	11	0.95	0.25	0.54
Ejin Banner	6	0.92	0.24	0.47	Jiuquan	12	0.96	0.21	0.51
Ejin Banner	7	0.98	0.23	0.54	Minqin	1	0.98	0.21	0.52
Ejin Banner	8	0.97	0.22	0.56	Minqin	2	0.92	0.20	0.51
Ejin Banner	9	0.91	0.20	0.52	Minqin	3	0.94	0.19	0.52
Ejin Banner	10	0.95	0.22	0.57	Minqin	4	0.94	0.24	0.54
Ejin Banner	11	0.91	0.26	0.53	Minqin	5	0.93	0.17	0.59
Ejin Banner	12	0.94	0.25	0.55	Minqin	6	0.89	0.19	0.51
Dunhuang	1	0.97	0.24	0.51	Minqin	7	0.88	0.24	0.56
Dunhuang	2	0.89	0.16	0.55	Minqin	8	0.94	0.24	0.54
Dunhuang	3	0.89	0.22	0.58	Minqin	9	0.93	0.23	0.58
Dunhuang	4	0.92	0.21	0.51	Minqin	10	0.89	0.24	0.59
Dunhuang	5	0.89	0.23	0.57	Minqin	11	0.90	0.18	0.58
Dunhuang	6	0.92	0.21	0.54	Minqin	12	0.91	0.21	0.51
Dunhuang	7	0.95	0.18	0.54	Gangcha	1	0.93	0.23	0.53
Dunhuang	8	0.93	0.24	0.55	Gangcha	2	0.91	0.24	0.62
Dunhuang	9	0.92	0.28	0.56	Gangcha	3	0.89	0.34	0.48
Dunhuang	10	0.90	0.23	0.48	Gangcha	4	0.93	0.36	0.54
Dunhuang	11	0.91	0.21	0.49	Gangcha	5	0.88	0.21	0.54
Dunhuang	12	0.90	0.23	0.52	Gangcha	6	0.89	0.24	0.56
Jiuquan	1	0.98	0.21	0.54	Gangcha	7	0.92	0.23	0.62
Jiuquan	2	0.98	0.22	0.52	Gangcha	8	0.89	0.30	0.55
Jiuquan	3	0.90	0.19	0.44	Gangcha	9	0.91	0.21	0.59
Jiuquan	4	0.95	0.27	0.55	Gangcha	10	0.93	0.23	0.48
Jiuquan	5	0.93	0.21	0.49	Gangcha	11	0.89	0.21	0.51
Jiuquan	6	0.96	0.20	0.54	Gangcha	12	0.88	0.21	0.59

**Table 5:** Statistics for the performance of the global daily solar radiation estimated by the proposed method at Jiuquan and Ejin Banner stations in 2008 (366 days).

Station	Angstrom Coefficients	Time	*R*^2^	*MBE* MJ·m^−2^·d^−1^	*MAE* MJ·m^−2^·d^−1^	*RMSE* MJ·m^−2^·d^−1^	*d*	*NSE*
Jiuquan	Monthly	2008.1.1–2008.12.31	0.9597	−0.1849	1.2432	1.6419	0.9873	0.9541
	Annual	2008.1.1–2008.12.31	0.9202	−0.8533	1.5853	2.3920	0.9723	0.9026
Ejin Banner	Monthly	2008.1.1–2008.12.31	0.9614	−0.3860	1.1291	1.4946	0.9894	0.9588
	Annual	2008.1.1–2008.12.31	0.9196	0.1035	1.5692	2.1187	0.9769	0.9172

**Table 6:** Statistics for the performance of daily net radiation estimated by the proposed method at the Arou, Yingke, Linze and Maliantan stations in 2008 (366 days).

Station	Sunshine Duration	Time	*R*^2^	*MBE* MJ·m^−2^·d^−1^	*MAE* MJ·m^−2^·d^−1^	*RMSE* MJ·m^−2^·d^−1^	*d*	*NSE*
Arou	FY-2D cloud-type	2008.1.1–2008.12.31	0.8703	1.0580	1.6908	2.1600	0.9518	0.8279
	Interpolation method	2008.1.1–2008.12.31	0.8232	1.8667	2.3700	2.8752	0.9172	0.6950
Yingke	FY-2D cloud-type	2008.1.1–2008.12.31	0.8903	0.5411	1.4519	1.8196	0.9684	0.8837
	Interpolation method	2008.1.1–2008.12.31	0.8533	1.5481	2.0199	2.5923	0.9454	0.7640
Linze	FY-2D cloud-type	2008.1.1–2008.12.31	0.9003	0.2162	1.2396	1.5925	0.9771	0.9160
	Interpolation method	2008.1.1–2008.12.31	0.7876	1.0041	1.9807	2.4724	0.9492	0.7975
Maliantan	FY-2D cloud-type	2008.1.1–2008.12.31	0.8489	1.9696	2.3552	2.8958	0.9231	0.7147
	Interpolation method	2008.1.1–2008.12.31	0.7559	2.0506	2.7992	3.3768	0.8892	0.6121
